# Effect of SiC Abrasive Blasting Parameters on the Quality of the Ceramic and Ni-Cr Dental Alloy Joint

**DOI:** 10.3390/ma15030964

**Published:** 2022-01-26

**Authors:** Emilia Wołowiec-Korecka, Weronika Czepułkowska-Pawlak, Zofia Kula, Leszek Klimek

**Affiliations:** 1 Institute of Materials Science and Engineering, Faculty of Mechanical Engineering, Lodz University of Technology, B. Stefanowskiego 1/15, 90-537 Lodz, Poland; weronika.czepulkowska@gmail.com (W.C.-P.); leszek.klimek@p.lodz.pl (L.K.); 2 Department of Dental Technology, Medical University of Lodz, Pomorska 251, 92-213 Lodz, Poland; zofia.kula@umed.lodz.pl

**Keywords:** abrasive blasting, metal-ceramic bond strength, shear strength, Ni-Cr alloy, thermocycles

## Abstract

The SiC abrasive blasting parameters are vital in ensuring a suitable bond between dental ceramics and the Ni-Cr alloy. The purpose of this in vitro test was to examine the strength of the joint between the Ni-Cr alloy and fused dental ceramics for SiC abrasive blasting at a specific pressure (400, 600 kPa) and particle size (50, 110, 250 µm) in order to determine the optimal treatment parameters. The test also accounted for thermal loads (5000 cycles, 5–55 °C) to which the metal-ceramic joint is subjected during use. One hundred and forty-four Ni-Cr cylinders were divided into six groups (*n* = 12) and subjected to the airborne-particle abrasion with SiC with various pressure and grit size parameters. After treatment, the specimens were rinsed, dried, fused to dental ceramics, and examined for their shear strength using the Zwick/Roell Z020 machine. The results were statistically analysed using the ANOVA analysis of variance (α = 0.05). The highest metal-ceramic joint strength was obtained for abrasive blasting with 110 and 250 µm SiC grit at a pressure of 400 kPa. This relationship was also observed after the joint was subjected to thermal loads (5000 thermocycles). Additionally, thermal loads did not significantly reduce the joint’s strength compared with non-loaded joints. For small SiC abrasive grit sizes (50 µm) under pressure 400 kPa, the treatment pressure had a significant effect on the strength of the joint (*p* < 0.05). For larger particle sizes, the pressure had no effect. After abrasive blasting using SiC, the Ni-Cr metal-ceramic joint retained its properties, even under thermal load, ensuring the joint properties’ stability during use.

## 1. Introduction

Ensuring a high bond strength between the metal substrate and dental ceramic in metal-ceramic prosthetic restorations is crucial in terms of their service life. Several factors contribute to the final bond strength, including the chemical bonds between the materials being joined and the stresses present in the bond caused by the difference in coefficients of thermal expansion (CTE) of materials [1,2]. However, it is the correct surface roughness obtained by abrasive blasting during the preparation of the metal surface for bonding to the ceramic which most significantly influences the durability of the joint. It provides points for mechanical anchoring of the ceramic during fusion [1,3].

Numerous studies have reported that the parameters used in abrasive blasting have different effects on the surface of metals used in prosthetics and on the strength of the metal-ceramic joint [3,4,5,6,7,8,9,10]. For titanium and cobalt-chromium alloys, the parameters that provide the best bond strength values include 110 µm aluminium oxide and a treatment pressure of 400 kPa [4,5], which contradicts the hypothesis that the higher the abrasive blasting parameters, the better the bond strength. Too small abrasive grit sizes or pressure values may result in insufficient surface roughness for the ceramic to flow into, so air is then easily trapped in the material boundary during the fusion of ceramic, which may not provide a permanent bond to the metal. Too large grit sizes or pressure values can result in irregularities that are too wide, which can also restrict bonding to the metal substrate [9]. Nickel-chromium alloys have long been used in dental prosthetics, mainly due to their favourable mechanical and technological properties. On the one hand, this allows the patient for a long-time and failure-free use the prosthetic restoration and, on the other, it facilitates the work of the dental technician who makes the restoration. In recent years, there has been a tendency of refraining from using alloys containing nickel due to the possibility of allergies in patients, associated mainly with the release of nickel ions from the alloy into the surrounding environment [11,12]. However, taking into account the favourable properties of these alloys, it seems that they can be used for prosthetic restorations on the condition of applying surface treatments that improve biocompatibility and reduce the possibility of allergy. Such research is conducted with coatings (oxide, carbon, nitride, carbide, etc.) used to cover the alloys of various metals [13,14,15,16,17]. The conducted research showed that the application of such coatings reduces the harmful effects of nickel, so these alloys can be confidently used after appropriate surface treatment [18,19,20,21]. The applied treatments will also allow for obtaining additional properties related to a reduction in bacterial biofilm formation and improvement of abrasion resistance or fretting wear, for example [22,23]. 

In prosthodontics, aluminium oxide (Al_2_O_3_) is widely used in abrasive blasting to create mechanical bonds between two materials. Silicon carbide (SiC) is a sharper abrasive grit and a harder material, so blasting may be more effective and the process more efficient. The aim of this study was to analyse the influence of different parameters of abrasive blasting using SiC grit on the strength of a metal-ceramic bond and to investigate the effect of thermal shocks on its durability.

## 2. Materials and Methods

One hundred and forty-four Heraenium^®^ NA nickel-chromium alloy samples (Heraeus Kulzer, Hanau, Germany) were cylinder-shaped with a diameter of 8 mm and a height of 15 mm. The chemical composition is presented in Table 1. The alloy’s chemical composition was determined by the X-ray fluorescence analysis method using an SRS300 spectrometer (SIEMENS, Berlin, Germany). Specimens were divided into two groups and were abrasive blasted (Alox 2001, Effegi Brega, Sarmato, Italy) using silicon carbide (SiC) for 20 s with a nozzle inclination of 45^°^ and a distance of 15 mm from the material surface. Each group of specimens was divided into six subgroups (*n* = 24). The groups were distinguished by abrasive blasting parameters where the variables were the abrasive grit size and the treatment pressure (Table 2). The abrasive blasted samples were processed in a commercial machine to abrasive blasted (Basic Master, Renfert, Hilzingen, Niemcy). After abrasive blasting, all specimens were cleaned in an ultrasonic cleaner (Emmi-55HC-Q, Emag, Oradea, Romania) in deionised water for 8 min to remove loose abrasive particles, and the surface was then dried with compressed air.

Then, IPS Classic^®^ dental ceramics (Ivoclar Vivadent, Schaan, Lichtenstein) were fused to such prepared surfaces in the form of two opaquer layers and two dentin layers according to the manufacturer’s recommendations (Table 3). The indicative thickness of the individual layers were 0.5, 0.5, and 2.0 mm, with the last layer complementing to 3 mm in order to ensure the appropriate possibility of testing the shear strength.

Each group was divided into two equally large subgroups. One subgroup in each group (*n* = 6) was subjected to thermal loads (thermocycles), including alternate immersion of the samples in baths of 5 °C and 55 °C. In practice, thermal loads occur during the consumption of cold meals (ice cream) with hot drinks (coffee). In the study, 5000 cycles of temperature changes were performed. The other subgroups were left intact. Specimens from all groups were then subjected to a shear strength test of the dental metal-ceramic bond (Zwick/Roell Z020, Zwick, Ulm, Germany). The specimens were loaded at a crosshead speed of 2 mm/min until failure. The results were statistically analysed using the Statistica statistical software (StatSoft, Tulsa, USA). A 3-factor ANOVA analysis of variance and a post-hoc Tukey Test were conducted (α = 0.05). After the shear strength tests were completed, the specimens were subjected to fractographic tests, which consisted of observations of the surfaces of obtained fractures in a scanning electron microscope JEOL JSM-6610LV (JEOL, Tokyo, Japan). In addition, the spatial distribution of elements was performed on the surfaces of the fractures to determine the nature and location of the joint fractures.

## 3. Results

The results of the shear strength tests of the Ni-Cr alloy-dental ceramic joint are shown in the table (Table 4). 

For joints not subjected to thermal loads, the highest shear strength values were observed for specimens blasted with the smallest abrasive grit size (50 µm) at a pressure of 400 kPa. For the pressure of 400 kPa, the trend of bond strength was decreasing as the SiC grit size increased, while for the pressure of 600 kPa, the trend was increasing. A significant difference in strength was observed in the group blasted with 50 µm particles at 400 kPa pressure compared with specimens blasted with 50 µm/600 kPa and 250 µm/400 kPa (*p* < 0.05). 

An important observation was made when comparing the strength of joints subjected to and not subjected to thermal loads (thermocycles). Thermal loads had a significant effect on the shear strength of the ceramic-metal joint (*p* < 0.05) (Table 4). 

**Figure 1:**
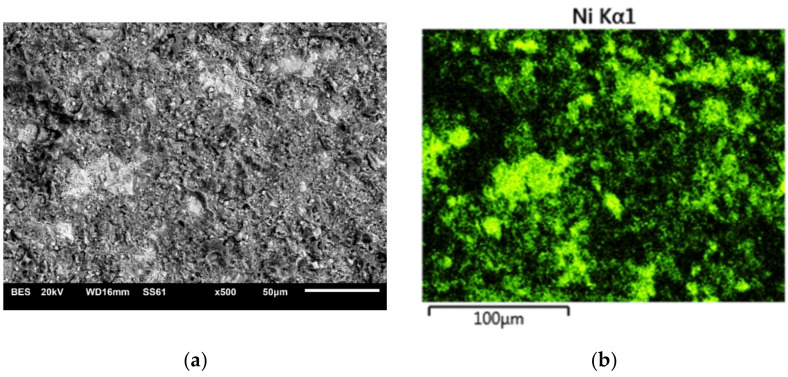

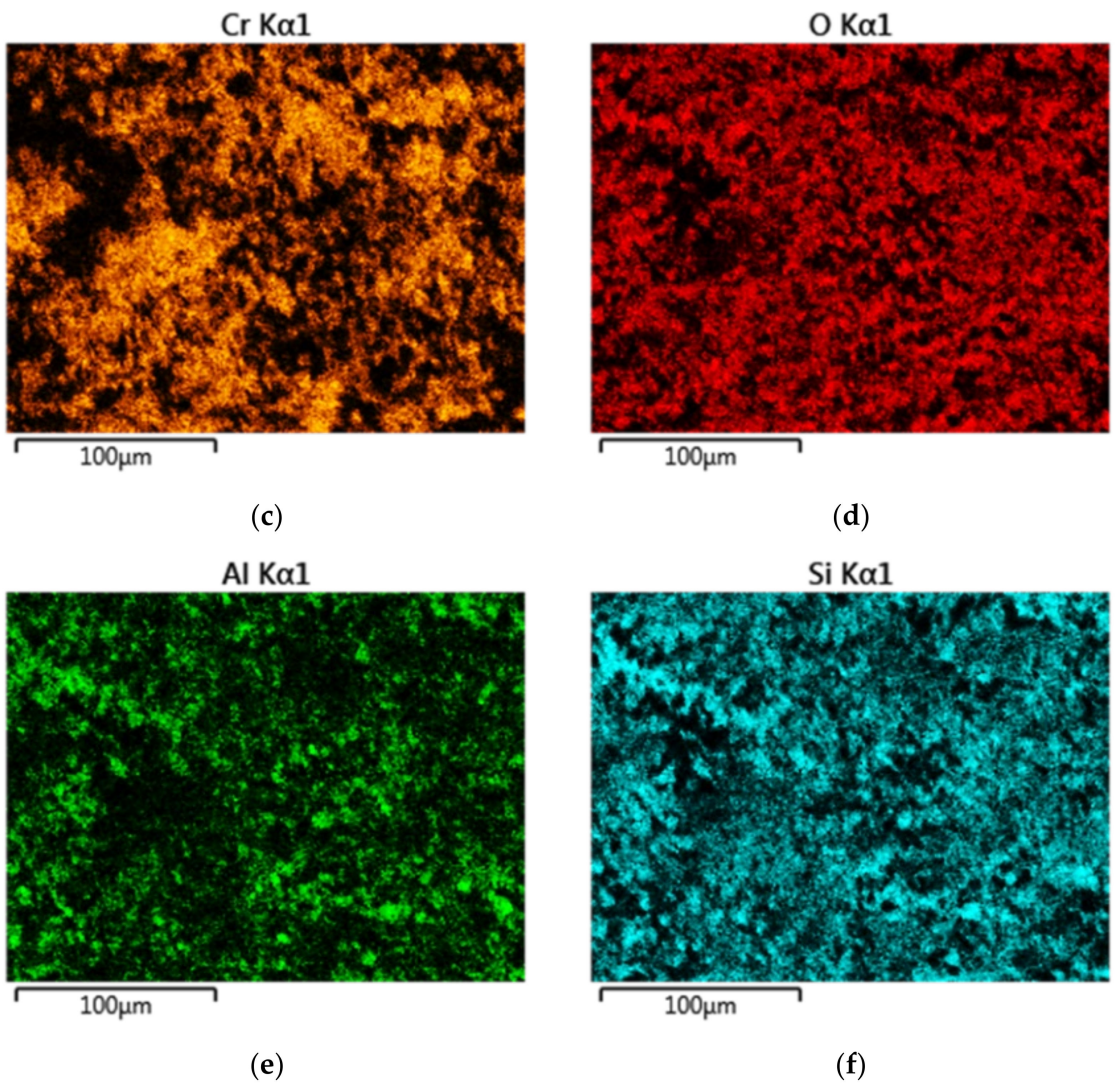
Microscopic image with the spatial distribution of elements in the fracture of S45 specimen (600 kPa/50 μm); (**a**) topography view; (**b**) surface distribution of nickel; (**c**) surface distribution of chromium; (**d**) surface distribution of oxygen; (**e**) surface distribution of aluminum; (**f**) surface distribution of silico.

**Figure 2:**
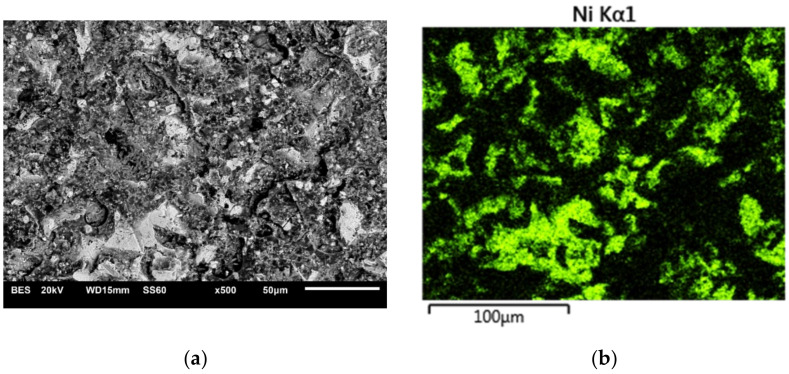

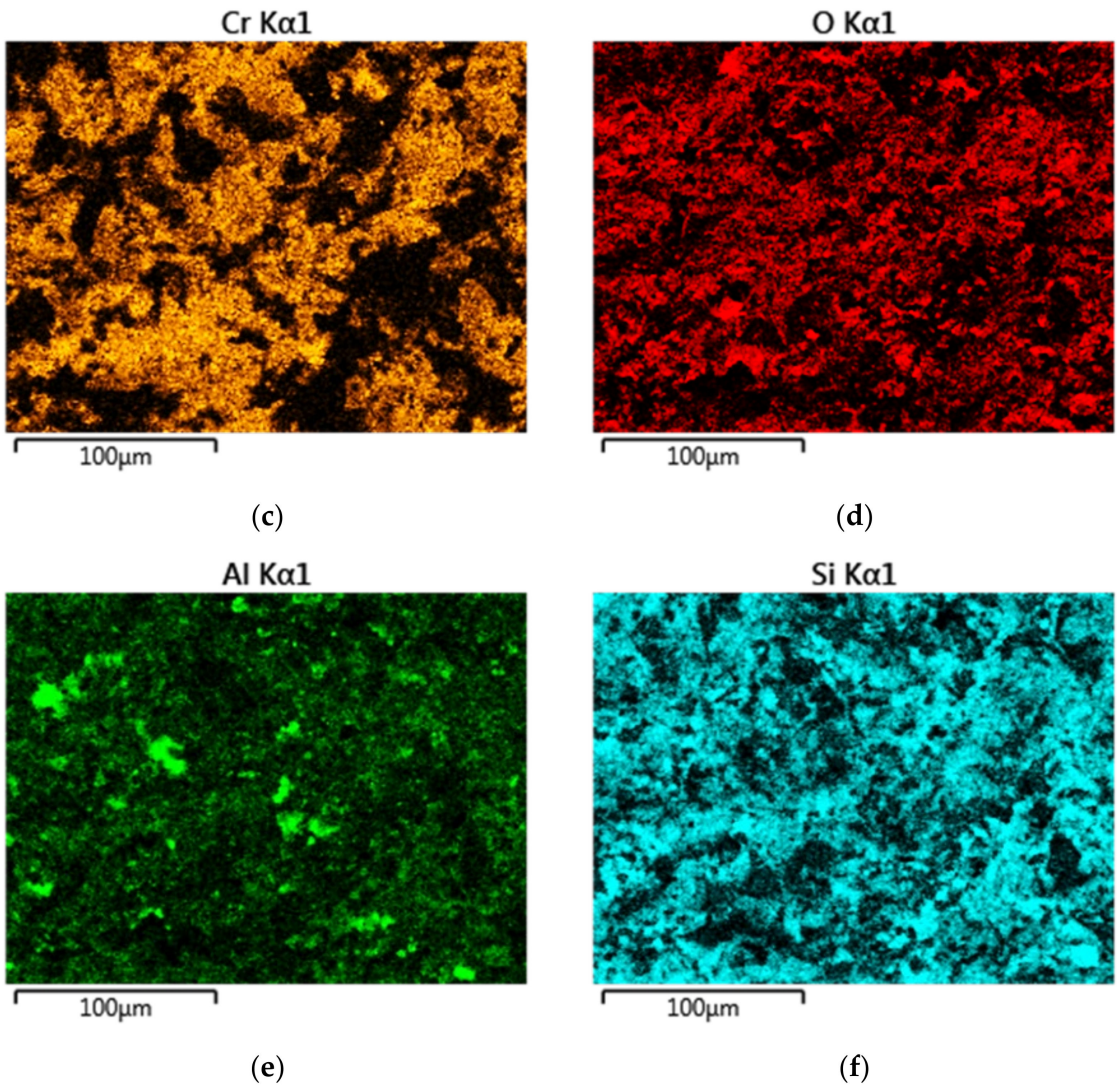
Microscopic image with the spatial distribution of elements in the fracture of S42 specimen (400 kPa/250 μm); (**a**) topography view; (**b**) surface distribution of nickel; (**c**) surface distribution of chromium; (**d**) surface distribution of oxygen; (**e**) surface distribution of aluminum; (**f**) surface distribution of silico.

The interaction “Pressure × Particle size” shown in Table 4 means that the grain size can significantly change the bonding strength only in conjunction with the pressure (specifically, for a grain size of 50 µm. During the treatment using this grain, the created bonding strength depends on the pressure). The figures (Figure 1 and Figure 2) show examples of fracture surface images along with the spatial distributions of elements.

The microscopic images of the fractures show both the elements contained in the metal substrate (Ni, Cr) and the ceramics (Si, Al). This indicates that during the shear test, the fracture occurred through both ceramics and metal, as well as, in some areas, at the interface between the metal substrate and veneering ceramics. The nature of the fractures in the shear test specimens after thermocyclic testing was analogous. 

## 4. Discussion

### 4.1. Pressure and Grit Size

The results of shear strength tests of the Ni-Cr alloy-dental ceramic joint showed a significant effect of the abrasive grain size in combination with pressure on the joint strength (Table 4). The highest strength values were obtained for the abrasive group blasted with 50 µm grain size at a pressure of 400 kPa. This is a new observation concerning research work on an Al_2_O_3_ abrasive [4,5,10,24]. Pietnicki et al. report that the optimum shear strength of the Co-Cr alloy and dental ceramics joint is provided after pre-treatment with an Al_2_O_3_ abrasive with a 110 µm grit size at a pressure of 400 kPa [4]. Similar results were published in a paper by Gołębiowski et al. that examined titanium-ceramic joints [5].

### 4.2. Thermal Loads

The conducted shear strength tests of the metal-ceramic joints after subjecting them to thermal loads (thermocycles) showed that the thermal load significantly influences the durability of the metal-ceramic bond. A similar relationship has been reported in publications for the Al_2_O_3_ abrasive, where a reduction in the bond strength after thermocycles has been observed for the bond between ceramics fused to a titanium [25,26,27,28], gold [26,29,30,31], or cobalt-chromium alloy [28,29,32]. Considering the above, when designing prosthetic restorations, it should be remembered that the strength of the metal-veneering ceramic bond may decrease during the use of such a restoration. The reason for the decrease in the strength of the alloy-ceramic joint and the elements subjected to cyclic thermal loads (thermocycles) can be explained by microcracks: as a result of cyclic heating and cooling of the element, strains develop in the joint-forming materials, which lead to microcracks weakening the joint (by reducing the actual cross-section of the joint) [25]. These strains are caused by differences in the coefficients of thermal expansion of the materials used for restorations (ceramic versus metallic alloy) [29]. Plasticity properties also play an important role in the occurrence and propagation of cracks. At an ambient temperature, nickel has a face-centred regular crystal lattice. This lattice has the largest amount of slip systems and deforms most easily, so stress relaxation by plastic deformation of the metal is most likely. In contrast, titanium and cobalt have a hexagonal lattice which has far fewer slip systems, so the possibility of relaxation by plastic deformation is lower. Differences in expansion coefficients play a major role in thermocycle-related cracking. However, this is not the only factor. The possibility of surface microcracking during abrasive blasting should also be considered. Abrasive grains hitting the workpiece surface can cause, apart from cutting, such micro-cracks. Silicon carbide, in comparison with aluminium oxide, is a material whose grains are more sharp-edged; therefore, it has a higher cutting ability. As a result, microcracks are less likely to be formed when hitting the workpiece surface and, consequently, there will be fewer micro-cracks than after treatment with aluminium oxide. 

### 4.3. Strength of the Metal—Dental Ceramic Bond

According to PN-EN ISO 9693:2020-04 [33], the minimum strength of an alloy-ceramic joint is 25 MPa; however, this is derived from the bending strength tests of the joint. In the case of the shear strength test, there are no standards specifying the minimum strength required, and there are no guidelines describing the appearance and size of the specimens. Therefore, it is not possible to directly compare the results of the tests carried out and the results provided for in the standard. The results measured after SiC abrasive blasting indicate joint strength values below 25 MPa; but, as said, due to the differences in the joint strength tests and in the shape and size of the specimens, it is not possible to compare the results of this study with the results from other research. 

### 4.4. Mechanism of the Metal—Dental Ceramic Bond Deterioration

Microscopic examination of the fractures showed that elements characteristic of both the alloy and the ceramic material were present on the surfaces of the nickel-chromium alloy. This may indicate a shear of adhesive-cohesive nature, which occurred both at the alloy-ceramic boundary and went through the ceramic itself. The fracture that occurred only in ceramic material was not observed, so it appears that the alloy-ceramic boundary is the weakest area of the bond. This is confirmed by bonding mechanisms, which mainly rely on mechanical anchorage and the occurrence of compressive stresses without the diffusion of elements between the materials. Under shear forces, the ceramic peels away from the surface. This exposes the substrate, and it occurs only at the anchorage points.

The described studies showed that the quality of the combination fares well in in vitro studies. However, it should be noted that it would be valuable to prepare a prosthetic restoration using the tested method and check its long-term behaviour in the patient’s cavity.

## 5. Conclusions

Based on the results presented in the study, the following conclusions were drawn:

1. Preparation of a metal-ceramic dental joint by blasting with SiC abrasives using a small grit size (50 µm) under pressure of 400 kPa results in optimal strength parameters of the joint (*p* < 0.05);
2. Thermal loads (thermal shocks) occurring during the use of the joint significantly reduce the strength of the joint (*p* < 0.05).

## Figures and Tables

**Table 1:** Chemical composition of the Heraenium^®^ NA alloy (wt.%).

Ni	Cr	Mo	Fe	Mn	Ta	Si	Co	Nb
residue	24.63	9.21	1.53	0.42	0.19	1.54	0.15	0.48

**Table 2:** The parameters of abrasive blasting processes.

SiC Abrasive Particle Size [µm]	Processing Pressure [kPa]	
	400	600
50	S45	S65
110	S41	S61
250	S42	S62

**Table 3:** The parameters of ceramic firing. V1—vacuum start temperature, V2—vacuum end temperature.

Layer No	Temp. (Max) [°C]	Resting Temp. [°C]	Drying Time [min]	Rise Temp. [°C]	Time [min]	V1 Temp. [°C]	V2 Temp. [°C]
Opaque							
I	980	403	6	80	1	550	979
II	970	403	6	80	1	550	969
Dentine							
I	920	403	4	60	1	580	919
II	910	403	4	60	1	580	909

**Table 4:** Results of the shear strength measurements of the Ni-Cr alloy-dental ceramic joint.

Pressure [kPa]	SiC Particle Size [µm]	Bond Strength [MPa] (Mean ± SD)		
		Without Thermocycles	After Thermocycles	Total (Pressure × Particle Size)
400	50	20.97 ± 4.12	18.28 ± 2.85	19.62 ± 3.73
400	110	17.57 ± 3.48	15.87 ± 3.95	16.72 ± 3.74
400	250	16.17 ± 3.64	16.04 ± 2.52	16.10 ± 3.06 ^(^*^)^
600	50	15.96 ± 3.66	13.83 ± 1.21	14.90 ± 2.88 ^(^*^)^
600	110	18.18 ± 2.93	16.61 ± 2.19	17.40 ± 2.65
600	250	19.03 ± 4.56	16.57 ± 2.62	17.80 ± 3.85
Total (Thermocycles)		17.98 ± 4.02 ^(A)^	16.20 ± 2.91 ^(B)^	
3-factor ANOVA				
Factor	F	*p*	Partial eta2	Power
Pressure	2.07	0.152	0.015	0.298
Particle size	0.11	0.895	0.002	0.067
Thermocycles	10.66	0.001	0.075	0.900
Pressure × Particle size	13.34	0.000	0.168	0.997
Pressure × Thermocycles	0.25	0.615	0.002	0.079
Particle size × Thermocycles	0.37	0.693	0.006	0.108
Pressure × Particle size × Thermocycles	0.68	0.507	0.010	0.163

* Significant difference compared with 50 µm/400 kPa; different letters indicate statistically significant difference.
